# Etiology and outcomes of acute kidney injury in Chinese children: a prospective multicentre investigation

**DOI:** 10.1186/1471-2490-13-41

**Published:** 2013-08-21

**Authors:** Yan Cao, Zhu-Wen Yi, Hui Zhang, Xi-Qiang Dang, Xiao-Chuan Wu, Ai-Wen Huang

**Affiliations:** 1 Division of Pediatric Nephrology, Children’s Medical Center, the Second Xiangya Hospital, Central South University, Changsha, Hunan 410011, P.R. China

**Keywords:** Acute kidney injury, Childhood, Multi-center, Clinical epidemiology, Investigation

## Abstract

**Background:**

The incidence of AKI appears to have increasing trend. Up to now, prospective, multi-center, large-sample epidemiological study done on pediatric AKI on aspects of epidemiological characteristics, causes and outcomes have not reported. It is necessary to develop prospective, multi-center, large-sample epidemiological study in our country on pediatric AKI. The aim of this study was to determine the clinical features, etiology, and outcomes of acute kidney injury (AKI) in Chinese children.

**Method:**

Paediatric patients (≤18 years old) admitted to 27 hospitals (14 children’s hospitals and 13 general hospitals) affiliated with the Medical University were investigated. AKI was defined using the 2005 Acute Kidney Injury Network criteria.

**Results:**

During the study period, 388,736 paediatric patients were admitted. From this total, AKI was diagnosed in 1,257 patients, 43 of whom died. The incidence and mortality of AKI was 0.32% and 3.4% respectively. The mean (± SD) age of patients was 48.4 ± 50.4 months. Among the 1,257 AKI paediatric patients, 632 were less than one year old. Among the AKI paediatric patients, 615 (48.9%) were in stage 1, 277 (22.0%) in stage 2, and 365 (29.0%) in stage 3. The most common causes of AKI were renal causes (57.52%), whereas postrenal (25.69%) and prerenal (14.96%) causes were the least common. The three most common causes of AKI according to individual etiological disease were urolithiasis (22.35%), of which exposure to melamine-contaminated milk accounted for the highest incidence (63.7%); acute glomerulonephritis (10.10%); and severe dehydration (7.48%). A total of 43 AKI patients (3.4%) died during their hospital stay; 15 (34.9%) of the 43 died as a result of sepsis.

**Conclusion:**

Primary renal diseases are a major risk factor for paediatric AKI in China. In terms of specific etiological disease, urolithiasis (postrenal disease) was the leading cause of paediatric AKI in 2008, when the disease was linked to exposure to melamine-contaminated milk. Sepsis is the leading cause of death in Chinese paediatric AKI patients. Future studies should focus on effective ways of controlling renal disorders and sepsis to improve the clinical management of paediatric AKI in China.

## Background

Studies showed that the development of acute kidney injury (AKI), defined as an acute increase in serum creatinine level (≥1.5x of baseline Scr or increase of 0.3 mg/dl of Scr in 48 hours), increases the risk of adverse outcomes in hospitalised patients [1-4]. The international kidney disease and critical care community recently proposed replacing the traditional term 'acute renal failure’ (ARF) with AKI because the latter closely reflects the pathophysiological nature of the disease. AKI is characterised by a reversible increase in blood concentration of creatinine and nitrogenous waste products as well as by the inability of the kidney to properly regulate fluid and electrolyte homeostasis. Renal injury can be divided into prerenal disease, renal disease including vascular insults, and postrenal disease. The prognosis of AKI is highly dependent on the underlying etiology of the AKI. Children who suffered AKI from any cause are at risk of developing kidney disease several years after the initial insult. The incidence of AKI in children appears to be increasing; in particular, the etiology of AKI in hospitalised children has shifted from primary renal disease to multifactorial causes over the past decades [1-3]. The survey conducted by Vachvanichsanong in Thailand from 1980 to 2004 showed that ARF incidence had a nearly ninefold increase in the PICU and in hospitalised children during the last 22 years [5]. The disease spectrum also changed significantly. A single-centre survey conducted from 1980 to 1990 [6] showed that haemolytic uremic syndrome (HUS), primary renal disease, sepsis, and burns are the main reasons for AKI in children. Recent studies showed significant changes in the epidemiological characteristics of AKI, the main causes of which include renal ischemia, the use of nephrotoxic drugs, and sepsis [7-9]. To date, no multicentre, large-sample epidemiological study has been conducted on paediatric AKI. Thus, we investigated the epidemiological characteristics of paediatric AKI at 27 hospitals in China. A better understanding of the etiology, incidence, and outcomes of paediatric AKI will help in designing effective strategies for early diagnosis and intervention of AKI. The epidemiology, common causes, and prognosis of AKI in hospitalised children in China are discussed in this paper.

## Methods

### Study population

Critically ill paediatric patients were enrolled from 14 provincial or municipal children’s hospitals and departments of paediatrics of 13 general hospitals affiliated with the Medical University all over China. Enrolment began on January 1, 2008 and was completed on December 31, 2008. This study was conducted in accordance with the Declaration of Helsinki. Ethical clearance was obtained from the Ethics Committee of Central South University, and written informed consents were obtained from the legal guardians of all the participants.

### Case screening

Cases were selected from paediatric inpatients (age ≤18 years old) in the Department of Nephrology, PICU, and NICU in children’s hospitals, and from paediatric inpatients of general hospitals. For patients who underwent multiple hospitalisations, the earliest dates of admission were the data considered in our final analysis.

### Inclusion criteria

AKI was diagnosed according to the criteria and protocols of the 2005 Acute Kidney Injury Network [10]. 1) The course of the disease is less than three months, with any abnormalities in blood, urine, and tissue attributable to the imaging findings of impaired renal structure and function. 2) An abrupt (within 48 hours) reduction in kidney function is defined as an absolute increase in serum creatinine of at least 0.3 mg/dl (≥26.4 μmol/l), a percentage increase in serum creatinine of at least 50% (1.5-fold from baseline), or a reduction in urine output (documented oliguria of less than 0.5 ml/kg per hour for more than 6 hours).

Exclusion criteria: 1) Children who were diagnosed with chronic kidney disease (CKD) on the first visit. 2) Children who were born with kidney injuries such as congenital malformations or birth defects. 3) Children with genetic metabolic diseases. 4) Children with known CKD whose AKI occurred during remission.

The classification/staging system for AKI was defined based on the following criteria [11]:

Stage 1: Increase in serum creatinine of at least 0.3 mg/dl (≥26.4 μmol/l), an increase of at least 150% to 200% (1.5- to 2-fold) from baseline, or urine output below 0.5 ml/kg per hour for more than 6 hours.

Stage 2: Increase in serum creatinine of more than 200% to 300% (>2- to 3-fold) from baseline or urine output of less than 0.5 ml/kg per hour for more than 12 hours.

Stage 3: Increase in serum creatinine of more than 300% (>3-fold) from baseline or serum creatinine of more than or equal to 4.0 mg/dl (≥354 μmol/l) with an acute increase of at least 0.5 mg/dl (44 μmol/l), or urine output less than 0.3 ml/kg per hour for 24 hours or anuria for 12 hours.

### Classification of etiologies of AKI

The standard approach used in this study to determine the etiologies of AKI utilised the following groupings: prerenal AKI caused by hypovolemia, decreased kinemia, vasodilation or renal artery contraction, which can lead to the decline of glomerular perfusion because of the decline of circulation volume without renal change; renal AKI caused by all kinds of kidney diseases including renal vascular, capillary, and glomerular diseases, acute interstitial nephritis, as well as acute tubular necrosis; and postrenal AKI seen in acute urinary obstruction, which is caused by numerous reasons but, by addressing the obstruction, can improve postrenal AKI.

### Clinical outcomes

Monthly follow ups for three months were conducted from the date of diagnosis. Subjects were divided into four groups based on the following clinical outcomes: Recovery (AKI indicators returned to normal and symptoms of primary disease disappeared), Improvement (AKI indicators returned to normal and symptoms of primary disease improved), CKD (condition persisted in the past three months with AKI indicators remaining abnormal), and Death.

### Survey methods

Questionnaires on demographic information such as age, gender, ethnicity, and date of admission were completed by trained nurses. All of the information were sent to the Pediatric Nephrology Department of Xiangya Second Hospital, Central South University. Clinical data, including primary diagnosis, AKI classification/staging, laboratory results, kidney imaging, renal pathology, outcomes, cause of death, and follow-up evaluation, were obtained from medical records.

### Statistical analysis

We calculated the mean ± standard deviations (x ± s) or the medians for continuous variables and proportion of categorical variables. T-test was used to compare the continuous variables of normal distributions. Rank sum test was utilised for the continuous variables with abnormal distributions. Chi-square test was employed to compare categorical variable data. *P* value <0.05 was considered significant. Data were analysed using SPSS11.5 software.

## Results

### Demographic information

Out of 388,736 paediatric patients admitted to the 27 hospitals during the study, 48,036 had kidney-related diagnoses (12.46%). A total of 1,257 patients met the inclusion criteria; 1,229 patients were Han, 15 were Zhuang, 5 were Manchu, 4 were Tujia, 3 were Hui, and 1 was Mongolian. Male-to-female ratio was 1.7:1, with 799 males (63.6%) and 458 females (36.4%), and mean age was 48.4 ± 50.4 months. The youngest patient was 15 days old and the oldest was 18 years old. One-year-old or younger patients (infants) were the most common, totalling 623 cases (49.6%) (Table 1). Average amount of time elapsed between onset of the disease and diagnosis of AKI was 5.5 ± 7.2 days. The 25%, 50%, 75%, 90%, and 95% values were 2, 3, 6, 11, and 18 days, respectively. A total of 279 (22.2%) patients came from cities while 978 (77.8%) came from rural areas.

**Table 1:** Age distribution of 1257 pediatric patients with AKI

	**Neonate**	**≤1**	**≈3**	**≈6**	**≈10**	**≈14**	**≈18**
Number of Cases	210	413	195	140	162	126	11
Proportion (%)	16.71	32.86	15.51	11.14	12.89	10.02	0.88

### Incidence of AKI

Of the 388,736 patients admitted, 1,257 were diagnosed with AKI. The overall incidence rate of AKI in the study population was 0.32% (95% CI: 0.30% to 0.34%). Among the1,257 patients with AKI, 615 cases (48.9%) were classified as stage 1, 277 cases (22.0%) as stage 2, and 365 cases (29.0%) as stage 3.

### Causes of AKI

The causes of AKI are presented in Table 2. Among the 1,257 AKI patients, 188 (14.96%) were pre-renal AKI, 723 (57.52%) were intrinsic AKI, 323 (25.69%) were postrenal AKI, and 23 (1.83%) had unknown causes. The most common causes were urolithiasis and acute glomerulonephritis (GN), followed by severe dehydration and nephrotic syndrome. Of the 281 urolithiasis patients, melamine was the cause for 179 cases (63.70%).

**Table 2:** Primary causes of pediatric AKI

**Primary disease**	**Cases**	**Constituent ratio (%)**	**Deaths**
**Prerenal AKI**	**188**	**14.96**	**4**
Severe dehydration	94	7.48	2
Neonatal blood loss	24	1.91	
Diarrhea	22	1.75	
Congenital heart disease	20	1.59	1
Significant blood loss	11	0.88	
Cardiomyopathy	9	0.72	1
Congenital hypertrophic pyloric stenosis	3	0.24	
Late vitamin K deficiency	2	0.16	
Infant muggy syndrome	2	0.16	
Myocarditis	1	0.08	
**Renal AKI**	**723**	**57.52**	**39**
Acute GN	127	10.10	
Nephrotic syndrome	78	6.21	3
Neonatal jaundice	67	5.33	
Sepsis	62	4.93	15
Drug poisoning	58	4.61	2
Henoch-Schonlein purpura	57	4.53	
Hemolytic uremic syndrome	43	3.42	1
Asphyxia	43	3.42	4
DIC	41	3.26	4
SLE	32	2.55	1
Pneumonia	26	2.07	6
Tumor	24	1.91	1
Acute interstitial nephritis	14	1.11	
Urinary tract infection	5	0.40	
IgA nephropathy/Berger’s disease	5	0.40	
HIE	5	0.40	
Traffic accident	5	0.40	
Hemolytic anemia	4	0.32	
Renal tubular acidosis	3	0.24	
Vesicoureteral reflux	3	0.24	
Epidemic hemorrhagic fever	3	0.24	
Renal vein thrombosis	3	0.24	
Intubation of umbilical cord	3	0.24	
Purulent meningitis	2	0.16	
Kawasaki disease	1	0.08	
Acute progressive GN	1	0.08	1
Drowning	1	0.08	1
Hemophagocytic syndrome	1	0.08	
CMV infection	1	0.08	
Hand-foot-mouth disease	1	0.08	
Tumor lysis syndrome	1	0.08	
Crush syndrome	1	0.08	
HBV-associated GN	1	0.08	
Xanthogranulomatous pyelonephritis	1	0.08	
**Postrenal AKI**	**323**	**25.69**	
Urolithiasis (179 by melamine)	281	22.35	
Congenital urinary tract malformations	42	3.34	
**Unknown**	**23**	**1.83**	

### AKI mortality

The hospital mortality of AKI was 3.4% (43 deaths) (95% CI: 2.7% to 4.1%). The most common cause of death was sepsis (15 cases, 34.9%), followed by pneumonia (6 cases, 14.0%). Of the 188 prerenal AKI patients, 4 died; mortality was 2.1%, (95% CI: 0.1% to 4.1%). There were 39 deaths among the 723 renal AKI cases, for a mortality rate of 5.4% (95% CI: 4.6% to 6.2%). No deaths occurred among the 323 postrenal AKI patients. Renal AKI had the highest fatality rate (÷^2^ = 3.54, *P* < 0.05). Among the 43 deaths, the imminent causes of death were septic shock (19 cases, 44.2%) and respiratory failure (8 cases, 18.6%) (Tables 3 and 4).

**Table 3:** The death rate observed among different causative diseases and constituent ratio in the 43 pediatric AKI death cases

**Primary disease**	**Cases**	**Deaths**	**Death rate (%)**	**Death constituent ratio (%)**
				**(n = 43)**
Prerenal AKI	188	4	2.1	9.3
Severe dehydration	94	2	2.1	4.7
Congenital heart disease	20	1	5.0	2.3
Cardiomyopathy	9	1	11.1	2.3
Renal AKI	723	39	5.4	90.7
Nephrotic syndrome	78	3	3.8	7.0
Sepsis	62	15	24.2	34.9
Drug poisoning	58	2	3.4	4.7
Hemolytic uremic syndrome	43	1	2.3	2.3
Asphyxia	43	4	9.3	9.3
DIC	41	4	9.8	9.3
SLE	32	1	3.1	2.3
Pneumonia	26	6	23.1	14.0
Tumor	24	1	4.2	2.3
Acute progressive GN	1	1	100	2.3
Drowning	1	1	100	2.3
Postrenal AKI	323	0	0	0

**Table 4:** The constituent ratio of causes of death as seen in 43 pediatric AKI death cases

**The causes of death**	**Cases**	**Constituent ratio (%)**
		**(n = 43)**
Septic shock	19	44.2
Respiratory failure	8	18.6
DIC	5	11.6
Renal failure	4	9.3
hypovolemic shock	2	4.7
Heart failure	2	4.7
Multiple organ failure	2	4.7
Pulmonary embolism	1	2.3

### Renal pathology

Pathological diagnosis of 125 patients who underwent renal biopsy revealed the following: endocapillary proliferative GN (36 cases, 28.8%), mesangial proliferative GN (19 cases, 15.2%), acute tubular necrosis and interstitial nephritis (12 cases, 9.6%), lupus nephritis (11 cases, 8.8%), purpura nephritis (10 cases, 8.0%), focal segmental glomerulosclerosis (7 cases, 5.6%), IgA nephropathy (7 cases, 5.6%), thrombotic microangiopathy (4 cases, 3.2%), acute tubular necrosis (4 cases, 3.2%), interstitial nephritis (3 cases, 2.4%), crescentic GN (2 cases, 1.6%), focal GN (2 cases, 1.6%), mesangial proliferative GN with acute tubular necrosis and interstitial nephritis (2 cases, 1.6 %), minor glomerular abnormalities (2 cases, 1.6 %), minimal change glomerular disease (2 cases, 1.6 %), sclerosing GN (1 case, 0.8%), and membrane proliferative GN (1 case, 0.8%).

### AKI outcome

Among the AKI patients, 809 (64.4%) recovered, 256 (20.4%) improved, 70 (5.6%) developed CKD, and 79 left treatment. A total of 190 patients accepted dialysis treatment; of these, 78 (41.1%) underwent intermittent hemodialysis, 65 (34.2%) underwent peritoneal dialysis, 22 (11.6%) underwent immune adsorption, 11 (5.8%) underwent continuous venovenous haemofiltration, 8 (4.2%) underwent plasma exchange, 5 (2.6%) underwent continuous venovenous haemodiafiltration, and 1 (0.5%) underwent slow continuous ultrafiltration (Table 5).

**Table 5:** Prognoses of 1257 acute kidney injury patients

**Outcome**	**Number of patients**	**Percentage (%)**
Recovery	809	64.4
Improvement	256	20.4
CKD	70	5.6
Death	43	3.4
Left therapy	79	6.3

## Discussion and conclusions

To the best of our knowledge, the present work is the most comprehensive study of its kind to determine the etiologies, characteristics, and outcomes of paediatric AKI using the current standard definition of AKI in China. No multicentre study of AKI exists that uses the standard definition with adults and children as subjects. Nevertheless, similar studies used definitions ranging from doubling of serum creatinine in relation to the requirement of dialysis. Our data showed that AKI remains a common clinical condition in hospitalised Chinese children. Paediatric AKI in China is associated with various clinical causes, with primary renal disorders as predominant causes. In addition, sepsis was found to be the leading cause of death in patients with paediatric AKI, with a high mortality rate.

Of the 1,257 children included in our study, 799 were males and 458 were females, resulting in an M:F ratio of 1.7:1, which is quite similar to the 1.8:1 ratio of other developing countries. In contrast, the M:F ratio in developed countries is 1:1 [12-14]. Kandoth showed that 67% of male children and only 23.5% of female children are brought to the hospital within 48 hours of oliguria. Our results showed that the majority of patients were from rural areas (77.8%).

AKI patients comprised 0.32% of the total number of hospitalised patients during the study period. This ratio ranged from 0.15% to 7.2% in other countries [15-17]. The prevalence of AKI varies considerably by region [18,19]. This finding may be related to the inconsistency in previous definitions of AKI and the diverse experiences of treatment and causes of AKI in different regions. Differences in the prevalence of AKI also depend largely on the distribution of primary disorders. One of the largest multicentre prospective studies, which included 29,269 cases in 54 ICUs in 23 countries, showed that the overall incidence of AKI was 5.7% [20].

This study found that the dominant type was renal AKI (57.52%). Urolithiasis, acute GN, severe dehydration, nephrotic syndrome, and neonatal jaundice were also major causes of AKI, and 63.7% of urinary stones in children were caused by melamine. Reports from other countries indicated that inadequate renal perfusion, surgery, and sepsis were the most common causes of AKI, but the spectra of AKI were different in developed countries from those of developing ones. Andreoli reported that the most common causes of AKI in children in developing countries are infection, acute GN, and HUS [21]. Hui–Stickle analysed 248 AKI patients in the Texas Children’s Hospital from 1998 to 2001 and found that the main causes of AKI were renal ischemic disease (21%), nephrotoxic drugs (16%), and sepsis (11%). Primary kidney disease and HUS accounted for 6.7% and 1.2% respectively [9]. Williams analysed 228 patients in medical institutions in Virginia from 1979 to 1998 and determined that the major causes of AKI were cardiac surgery, sepsis, blood cancer, and respiratory failure [8]. The disease spectrum of AKI also changed over time. Srivastava evaluated 174 cases of AKI in a medical centre in northern India from 1972 to 1979 and discovered the causes of AKI to be acute gastroenteritis (25%), post-infectious GN (23%), and intravascular hemolysis (11%) [22]. Ten years later, 205 AKI patients in the same centre showed major causes of HUS (36%), post-infectious GN (17%), nephritis (13%), and intravascular hemolysis (6%) [23]. Children with lithangiuria were rare in the past. However, our study showed that lithangiuria is now one of the leading causes of AKI, and that 66.9% of the cases were children less than one year old (Table 6). The unfortunate incident that occurred in China in September 2008 was a global issue. Among 281 lithangiuria patients, 179 (63.7%), mostly infants, were fed with melamine-contaminated milk for a long time. This resulted in multiple stones, which were small in size, low in density, and shaped in various ways. However, the majority of casualties were outpatients and those who were not admitted to hospitals.

**Table 6:** The distribution of 1257 pediatric AKI cases of different causative disease according to age

**Primary disease**	**Neonate**	**≤1**	**≈3**	**≈6**	**≈10**	**≈14**	**≈18**
	**Cases**	**Cases**	**Cases**	**Cases**	**Cases**	**Cases**	**Cases**
**Prerenal disease**	43	108	22	13	2	0	0
Severe dehydration	13	81	0	0	0	0	0
Neonatal blood loss	24	0	0	0	0	0	0
Diarrhea	0	4	9	7	2	0	0
Congenital heart disease	4	8	7	1	0	0	0
Significant blood loss	2	4	3	2	0	0	0
Cardiomyopathy	0	4	3	2	0	0	0
Congenital hypertrophic pyloric stenosis	0	3	0	0	0	0	0
Late vitamin K deficiency	0	2	0	0	0	0	0
Infant muggy syndrome	0	2	0	0	0	0	0
Myocarditis	0	0	0	1	0	0	0
**Renal disease**	152	85	100	101	154	120	11
Acute GN	0	0	18	18	66	24	1
Nephrotic syndrome	0	0	26	17	17	13	5
Neonatal jaundice	67	0	0	0	0	0	0
sepsis	4	22	19	12	2	3	0
Drug poisoning	5	27	15	8	2	1	0
Henoch-Schonlein purpura	0	0	0	3	25	25	4
Hemolytic uremic syndrome	0	0	0	11	11	21	0
Asphyxia	43	0	0	0	0	0	0
DIC	25	16	0	0	0	0	0
SLE	0	0	0	0	16	16	0
Pneumonia	0	11	4	11	0	0	0
tumor	0	0	4	4	7	9	0
Acute interstitial nephritis	0	0	4	6	2	2	0
Urinary tract infection	0	1	3	1	0	0	0
IgA nephropathy/Berger’s disease	0	0	0	0	3	2	0
HIE	5	0	0	0	0	0	0
Traffic accident	0	0	2	3	0	0	0
Hemolytic anemia	0	0	1	0	2	1	0
Renal tubular acidosis	0	1	0	2	0	0	0
Vesicoureteral reflux	0	0	0	2	1	0	0
Epidemic hemorrhagic fever	0	0	0	2	0	0	1
Renal vein thrombosis	0	3	0	0	0	0	0
Intubation of umbilical cord	3	0	0	0	0	0	0
Purulent meningitis	0	1	1	0	0	0	0
Kawasaki disease	0	0	0	1	0	0	0
Acute progressive GN	0	0	0	0	0	1	0
drowning	0	0	1	0	0	0	0
Hemophagocytic syndrome	0	0	1	0	0	0	0
CMV infection	0	1	0	0	0	0	0
Hand-foot-mouth disease	0	1	0	0	0	0	0
Tumor lysis syndrome	0	0	0	0	0	1	0
Crush syndrome	0	1	0	0	0	0	0
HBV-associated GN	0	0	0	0	0	1	0
Xanthogranulomatous pyelonephritis	0	0	1	0	0	0	0
**postrenal disease**	15	220	73	26	6	6	0
Urolithiasis (179 by melamine)	0	188	56	26	6	5	0
Congenital urinary tract malformations	15	19	7	0	0	1	0
unknown	0	13	10	0	0	0	0

The kidney is one of the vital organs associated with drug metabolism and excretion. Drug-induced kidney injury is a main cause of acute renal failure. In this study, drug-related AKI accounted for 4.1% of all AKI cases, significantly lower than the 19.9% reported by the Nephrology Department of Ruijin Hospital at the Shanghai Jiaotong University School of Medicine [24]. In our study, antibiotics resulted in 17 cases (29.3%). Specifically, aminoglycosides resulted in 5 cases (8.6%), cephalosporin V resulted in 4 (6.9%), cephalosporin VI and sulfonamides resulted in 3 each (5.2%), and vancomycin (1.7%) and rifampicin resulted in 1 each (1.7%). Fourteen cases of AKI were caused by anticancer drugs (24.1%), 7 by anti-inflammatory drugs (12.1%), and 7 cases by contrast agents (12.1%). A cohort showed that the most common causes were non-steroidal anti-inflammatories and gentamicin [25]. In our cohort, 29.3% of the AKI patients were exposed to antibiotics, a figure that highlighted the unsolved and alarming issue on overuse of antibiotics in China. This rationalises the urgency of standardising the use of antibiotics.

In the case of toddlers, our data showed that all 6 cases of traffic accident and drowning happened among children between 1 and 6 years old (Table 6). Thus, protection against accidents is imperative.

In our study, the mortality rate of AKI patients was 3.4%, significantly lower than the 20% to 41.5% reported in previous studies [9,26-28]. Furthermore, the main causes of AKI were urinary tract stones, acute nephritis, and nephrotic syndrome. The diseases responsible for the highest mortality rates, such as sepsis, malignant tumours, and HUS, accounted for a relatively slight proportion of the cases of AKI. Septic AKI was the most common cause of death, accounting for 34.9%. Sepsis is common in ICUs, particularly in patients with AKI. The mortality rate of septic AKI is reported to be as high as 74.5% [18]. Data show that sepsis is not the main primary disease that causes AKI, but it is the most important cause of death among AKI patients. Among 62 cases of sepsis, 57 were 6 years and below. This situation indicates that emphasis should be placed on sepsis, specifically in the case of young children, and on the need for further research on septic AKI. Gradual collection of clinical epidemiology data can lead to the development of control strategies that can improve clinical diagnosis and treatment. The AKI concepts presented by this study are expected to raise awareness on early kidney damage in critically ill patients and improve early intervention to prevent the occurrence of ARF. These actions may reduce the mortality rate.

The limitations of our study are as follows. First, we selected patients from the Department of Nephrology, PICU, and NICU in children’s hospitals and general hospitals, but disregarded the patients in other departments, which caused selection bias. The referral of paediatric patients scheduled for surgery to the adult departments of general hospitals was a challenge in terms of information collection. Nevertheless, the patients we selected provided highly relevant results in relation to AKI. Thus, our multicentre survey results can provide information about the incidence and outcome of AKI. Second, the management of patients was not standardised in various centres. In most cases, treatment of the disease was based on personal experience. Third, selection of subjects failed to consider patients who may have died before being diagnosed with AKI. This situation was difficult to avoid.

We conducted a large multicentre observational study of the incidence and outcomes of paediatric AKI in a large, heterogeneous population in China. Our data revealed that AKI is a common and important clinical issue in children; thus, attention should be given to the prevention and treatment of primary diseases, such as primary renal disease, severe dehydration, sepsis, and urinary tract stones, as well as those caused by nephrotoxic drugs. Since septic AKI was identified as the main cause of death, the effective control of sepsis may help to improve paediatric AKI outcomes.

## Competing interests

All authors have no conflict of interest regarding this paper.

## Authors’ contributions

Z-WY and YC designed the study; Children’s AKI Survey Collaborative Group performed the study; YC and HZ analyzed the data; YC and Z-WY wrote the paper. All authors read and approved the final manuscript.

## Pre-publication history

The pre-publication history for this paper can be accessed here:

http://www.biomedcentral.com/1471-2490/13/41/prepub

## AKI Survey Collaborative Group participants (No particular order)

The Second Xiangya Hospital, Central South University (Yan Cao, Zhu-wen Yi, Hui Zhang, Xi-qiang Dang, Xiao-chuan Wu, Ai-wen Huang); Beijing Children’s Hospital, Capital Medical University (Ying Shen, Zhi-hui Liu); Children's Hospital of Fudan University(Hong Xu, Hai-rong Gong); Children's Hospital of Shanghai (Guan-hua Zhu), The First Affiliated Hospital of Sun Yat-sen University (Xiao-yun Jiang); Tongji Hospital of Huazhong Science and Technology University (Jian-hua Zhou); The First Affiliated Hospital of China Medical University (Hong Jiang); Shengjing Hospital of China Medical University (Yu-bin Wu); the First Affiliated Hospital of Guangxi Medical University (Yuan-han Qin); Wuhan City Children's Hospital (Jiang-wei Luan); Hunan Provincial Children's Hospital (Zhi-hui Li, Zheng-hui Xiao); Children's Hospital, Zhejiang University School of Medicine (Ai-min Liu); Children's Hospital Affiliated to Suzhou University (Yue Min, Xiao-zhong Li); Children's Hospital of Jiangxi Province (Bao-jin Hu); Anhui Provincial Children's Hospital (Yang Dong, Yao-hui Zhang); Guangzhou Children's Hospital (Yan Gao); Nanjing Children's Hospital (Song-ming Huang); Xi'an Children's Hospital (Ying Bao); Hainan Provincial People's Hospital (Wei Xiang, Guo-zhen Zhang); Guangzhou Municipal First People's Hospital (Li Yu); First Affiliated Hospital of Anhui Medical University (Bo Hu, Ling Lu); Zhengzhou Children’s Hospital (Cui-hua Liu); First Affiliated Hospital of Lanzhou University (Yu Jin, Yu-ning Li); The First Affiliated Hospital of Xinxiang Medical College (Zi-ming Han, Ling-yun Bi); Wenzhou Medical College, Yuying Children's Hospital (Qing Yang); Fuzhou General Hospital of Nanjing Military Region (Zi-hua Yu, Jun Huang); Xuzhou Medical College Affiliated Hospital (Feng-jun Guan).

## References

[B1] Lassnigg A Schmidlin D Mouhieddine M Bachmann LM Druml W Bauer P Hiesmayr M Minimal changes of serum creatinine predict prognosis in patients after cardiothoracic surgery: a prospective cohort study J AM soc Nephrol 2004 15 1597 1605 10.1097/01.ASN.0000130340.93930.DD 15153571

[B2] Akcay A Turkmen K Lee D Edelstein CL Update on the diagnosis and management of acute kidney injury Int J Nephrol Renovasc Dis 2010 3 129 140 21694939 10.2147/IJNRD.S8641 PMC3108768

[B3] Coca SG Yusuf B Shlipak MG Garg AX Parikh CR Long-term risk of mortality and other adverse outcomes after acute kidney injury: a systematic review and meta-analysis Am J Kidney Dis 2009 53 961 973 10.1053/j.ajkd.2008.11.034 19346042 PMC2726041

[B4] Wamoch DG Towards definition and classification of acute kidney injury J Am Soc Nephrol 2005 16 3149 3150 10.1681/ASN.2005090934 16207828

[B5] Vachvanichsanong P Dissaneewate P Lim A McNeil E Childhood acute renal failure: 22-year experience in a university hospital in southern Thailand Pediatrics 2006 118 e786 e791 10.1542/peds.2006-0557 16894011

[B6] Andreoli SP Acute renal failure Curr Opin Pediatr 2002 14 183 188 10.1097/00008480-200204000-00007 11981288

[B7] Flynn JT Choice of dialysis modality for management of pediatric acute renal failure Pediatr Nephrol 2002 17 61 69 10.1007/s004670200011 11793137

[B8] Williams DM Sreedhar SS Mickell JJ Chan J Acute kidney failure: a pediatric experience over 20 years Arch Pediatr Adolesc Med 2002 156 893 900 10.1001/archpedi.156.9.893 12197796

[B9] Hui-Stickle S Brewer ED Goldstein SL Pediatric ARF Epidemiology at a Teritary Care Center from 1999 to 2001 Am J Kidney Dis 2005 45 96 101 10.1053/j.ajkd.2004.09.028 15696448

[B10] Ronco C Levin A Warnock DG Mehta R Kellum JA Shah S Molitoris BA Improving outcomes from acute kidney injury (AKI): Report on an initiative Int J Artif Organs 2007 30 373 376 17551899

[B11] Abuelo JG Normotensive ischemic acute renal failure N Engl J Med 2007 357 797 805 10.1056/NEJMra064398 17715412

[B12] Kandoth PW Agarwal GJ Dharnidharka VR Acute renal failure in children requiring dialysis therapy Indian Pediatr 1994 31 305 309 7896366

[B13] Phadke KD Dinakar C The challenges of treating children with renal failure in a developing country Perit Dial Int 2001 21 Suppl 3 S326 S329 11887846

[B14] Agarwal I Kirubakaran C Markandeyulu V Clinical profile and outcome of acute renal failure in South Indian children J Indian Med Assoc 2004 102 353 354 15717579

[B15] Lameire N Van Biesen W Vanholder R The changing epidemiology of acute renal failure Nat Clin Pract Nephrol 2006 2 364 377 16932465 10.1038/ncpneph0218

[B16] Obialo CI Okonofua EC Tayade AS Riley LJ Epidemiology of de novo acute renal failure in hospitalized African Americans: comparing community-acquired vs hospital-acquired disease Arch Intern Med 2000 160 1309 1313 10.1001/archinte.160.9.1309 10809034

[B17] Nash K Hafeez A Hou S Hospital-acquired renal insufficiency Am J Kidney Dis 2002 39 930 936 10.1053/ajkd.2002.32766 11979336

[B18] Lameire N Van Biesen W Vanholder R Acute renal failure Lancet 2005 365 417 430 15680458 10.1016/S0140-6736(05)17831-3

[B19] Lameire N Van Biesen W Vanholder R The rise of prevalence and the fall of mortality of patients with acute renal failure: what the analysis of two databases does and does not tell us J Am Soc Nephrol 2006 17 923 925 10.1681/ASN.2006020152 16540555

[B20] Uchino S Kellum JA Bellomo R Doig GS Morimatsu H Morgera S Schetz M Tan I Bouman C Macedo E Gibney N Tolwani A Ronco C Acute renal failure in critically ill patients: a multinational, multicenter study JAMA 2005 294 813 818 10.1001/jama.294.7.813 16106006

[B21] Andreoli SP Acute kidney injury in children Pediatr Nephrol 2009 24 253 263 10.1007/s00467-008-1074-9 19083019 PMC2756346

[B22] Srivastava RN Choudhry VP Acute renal failure in Delhi Indian J Pediatr 1982 49 65 70 7106928

[B23] Srivastava RN Bagga A Moudgil A Acute renal failure in north Indian children Indian J Med Res 1990 92 404 408 2079354

[B24] Ma J Zhang W Qin L Shen PY Xu YW Ren H Pen XX Zhu P Chen XN Chen N Drug-induced acute kidney injury: a clinical and pathological analysis Shanghai Medical Journal 2009 32 214 217

[B25] Li S Krawczeski CD Zappitelli M Devarajan P Thiessen-Philbrook H Coca SG Kim RW Parikh CR Incidence, risk factors, and outcomes of acute kidney injury after pediatric cardiac surgery–a prospective multicenter study Crit Care Med 2011 39 1493 1499 10.1097/CCM.0b013e31821201d3 21336114 PMC3286600

[B26] Askenazi DJ Feig DI Graham NM Hui-Stickle S Goldstein SL 3–5 year longitudinal follow up of pediatric patients after acute renal failure Kidney Int 2006 69 184 189 10.1038/sj.ki.5000032 16374442

[B27] Bailey D Phan V Litalien C Ducruet T Mérouani A Lacroix J Gauvin F Risk factors of acute renal failure in critically ill children: A prospective descriptive epidemiological study Pediatr Crit Care Med 2007 8 29 35 10.1097/01.pcc.0000256612.40265.67 17251879

[B28] Gokcay G Emre S Tanman F Sirin A Elcioglu N Dolunay G An epidemiological approach to acute renal failure in children J Trop Pediatr 1991 37 191 193 10.1093/tropej/37.4.191 1960778

